# Continuity and coordination of care in highly selected chronic cancer patients treated with multiple repeat radiation therapy

**DOI:** 10.1186/s13014-021-01949-5

**Published:** 2021-11-24

**Authors:** Sebastian M. Christ, Maiwand Ahmadsei, Annina Seiler, Eugenia Vlaskou Badra, Jonas Willmann, Caroline Hertler, Matthias Guckenberger

**Affiliations:** 1 Department of Radiation Oncology, University Hospital Zurich, University of Zurich, Rämistrasse 100, 8091 Zurich, Switzerland; 2 Competence Center for Palliative Care, University Hospital Zurich, University of Zurich, Zurich, Switzerland

**Keywords:** Continuity of care, Radiation therapy, Repeat irradiation, Chronic cancer disease

## Abstract

**Introduction and background:**

As cancer is developing into a chronic disease due to longer survival, continuity and coordination of oncological care are becoming more important for patients. As radiation oncology departments are an integral part of cancer care and as repeat irradiation becomes more commonplace, the relevance of continuity and coordination of care in operating procedures is increasing. This study aims to perform a single-institution analysis of cancer patients in which continuity and coordination of care matters most, namely the highly selected group with multiple repeat course radiotherapy throughout their chronic disease.

**Materials and methods:**

All patients who received at least five courses of radiotherapy at the Department of Radiation Oncology at the University Hospital Zurich from 2011 to 2019 and who were alive at the time of the initiation of this project were included into this study. Patient and treatment characteristics were extracted from the hospital information and treatment planning systems. All patients completed two questionnaires on continuity of care, one of which was designed in-house and one of which was taken from the literature.

**Results:**

Of the 33 patients identified at baseline, 20 (60.6%) participated in this study. A median of 6 years (range 3–13) elapsed between the first and the last visit at the cancer center. The median number of involved primary oncologists at the radiation oncology department was two (range 1–5). Fifty-seven percent of radiation therapy courses were preceded by a tumor board discussion. Both questionnaires showed high levels of experienced continuity of care. No statistically significant differences in experienced continuity of care between groups with more or less than two primary oncologists was found.

**Discussion and conclusion:**

Patients treated with multiple repeat radiation therapy at our department over the past decade experienced high levels of continuity of care, yet further efforts should be undertaken to coordinate care among oncological disciplines in large cancer centers through better and increased use of interdisciplinary tumor boards.

**Supplementary Information:**

The online version contains supplementary material available at 10.1186/s13014-021-01949-5.

## Introduction and background

As cancer is developing into a chronic disease, continuity and coordination of oncological care are becoming more important. Continuity and coordination of care are broad and often only loosely defined concepts which intend to capture the integration of patient care over time [1]. Continuity of care is often taken to refer to the patient/”subjective” view, whereas the coordination of care is supposed to reflect the provider/”objective” view [2]. The subjective component is best measured as a patient reported experience measure (PREM) [3]. The objective component can be evaluated with the help of different care metrics. Continuity of care has been separated into three parts in the literature: *informational*, *managerial* and *relational* aspects [4]. Informational continuity refers to the inclusion of a patient’s past medical history and all relevant individual circumstances when making decisions about a patient’s current care need [4]. Managerial continuity means a holistic and well-organized diagnostic and therapeutic scheme in which a patient is managed over time [4]. And relational continuity is used to describe the building of a lasting relationship between a patient and the involved healthcare providers [4]. Coordination of care has been separated into two subcategories, *management coordination*—good collaboration between care providers—and *information coordination*—the unseeming and coordinated flow and use of information regarding patient cases [5].

The beneficial effect of the continuity and coordination of care has been demonstrated for various specific patient populations [6, 7]. There is evidence that case mortality is lower when cancer patients receive treatment at comprehensive cancer centers where treating physicians are familiar with the patient history [8]. Better continuity of care is positively associated with improved symptom palliation and supportive care [9]. Continuity and coordination of care have also been shown to lead to an improved patient experience, as it enables healthcare providers to focus more on the patient rather than on the disease [10]. Continuity and coordination of care was shown to be a major contributor to patient satisfaction [11]. In addition, there is an indication that continuity of care in cancer patients improves when less rather than more health care professionals as well as integrated palliative care teams are involved [12]. Continuity and coordination of care have also been of increasing interest to healthcare policy makers, as they look at streamlining care pathways of patients, which include various clinical specialties at large comprehensive cancer centers where high staff turn-over not uncommon [13].

Clinical cancer care pathways are known to be amongst the most difficult to manage [14]. As cancer histologies are being broken down into more and more sub-groups, cancer patients are being confronted with a growing numbers of medical subspecialists and subspeciality care providers [5]. Of all patients diagnosed with cancer, every second will receive radiotherapy (RT) during their course of disease [15]. Hence radiation oncology departments constitute an integral part of all comprehensive cancer centers globally. The overwhelming majority of patients presenting for treatment at a radiation oncology department, however, will only receive RT once during their disease course or even lifetime [16]. Yet as cancer is becoming a chronic condition, and as more and more patients outlive their diagnosis by many years, the probability of frequenting radiation oncology departments multiple times and receiving multiple radio-oncological treatments increases in parallel.

The aim of this study is to explore aspects of continuity and coordination of care in the highly selected and small group of patients who were treated with multiple courses RT at the Department of Radiation Oncology at the University Hospital Zurich (USZ) for their cancer diagnosis during an extended period of time of their chronic cancer disease. As radiation oncology department at a leading university hospital in Switzerland, our department functions as an integral part of the Comprehensive Cancer Center Zurich (CCCZ). Though this is a single-center analysis, we hope it will inspire other radiation oncology departments to conduct similar studies, so that pooled-data or multi-center studies will be possible in the future to assess and improve continuity of care for radio-oncological patients.

## Materials and methods

### Patient population

All patients included in this study received at least five courses of RT at the Department of Radiation Oncology of the USZ within the last decade. This large number of RT courses was chosen to investigate the most complex cohort of patients with the most treatments and year-long follow-up, where continuity and coordination of care appear most important. A cut-off of two or three RT treatments seemed too low to adequately assess continuity and coordination of care over time, which is why a minimum of five RT courses was chosen. A RT course was defined as a prescribed RT regimen to one anatomical site for one medical indication. To identify this patient cohort, we screened all treatment records in the Record and Verify System (Aria® Version 15, Varian®) to long-list patients who received at least five RT courses between 2011 and 2019. We used the term *multiple repeat RT* (MRRT) to characterize the treatment of such patients. A short-list of patients was then obtained by selecting patients who were still alive at the time of study design and initiation in fall 2020.

### Electronic patient records

Treatment variables were extracted from the treatment planning system ARIA® and demographic and disease variables from the hospital information system (HIS) KISIM™. Treatment parameters included treatment site, RT course identifier, plan name, RT start date, RT end date, RT fractionation schedule, single dose, total dose and course count. Demographic and disease parameters comprised unique patient identifier, gender, date of birth, survival status, primary diagnosis, name of primary oncologists (PO) per prescribed RT course, list of clinically involved departments at USZ in the patient-specific cancer care pathway, clinical department with follow-up lead and involvement of a multidisciplinary tumor board (MDT) in RT recommendation per RT course. PO was defined as the radiation oncologist who consulted the patient, prescribed treatment, contoured and signed off on the delineation of the target volumes and organs at risks, approved the treatment plans and regularly monitored the RT. Clinically involved department was defined as regularly patient-facing, resulting in pathology, radiology and nuclear medicine departments being excluded from the head count of involved disciplines, even though they hold a key role for every cancer case under discussion and treatment at CCCZ. Clinical department with follow-up lead was defined as the department who remained in most regular contact with the patient, determined clinical and imaging follow-up schedules, initiated MDT discussions, communicated new findings to the patient and directed next therapeutic steps. MDT involvement was defined as a patient case having been presented and discussed prior to a new RT course.

### Employed measures for continuity and coordination of care

The objective component of continuity and coordination of care was defined as (1) the number of different POs at our department which managed radio-oncological care over time; (2) the number of RT courses preceded by an MDT as surrogate of the coordination of care amongst various involved clinical departments. The proxy variables for measuring the objective components of continuity and coordination of care were manually extracted from the HIS as described above.

To capture the subjective patient-reported component, we used two questionnaires, (1) one in-house developed questionnaire, consisting of ten questions and developed to address psycho-oncological, palliative and relational aspects (Additional file 1: Questionnaire 1), and (2) one validated questionnaire with 17 questions from the literature [17] (Additional file 1: Questionnaire 2). The in-house developed questionnaire was created to capture psycho-oncological, palliative and the relational aspect of the continuity of care within our department. It was developed by a team consisting of radiation oncologists, an experienced psycho-oncologist as well as a senior palliative care specialist. Patients were asked to answer eight questions on a four-point Likert scale: 1 = does not apply, 2 = does rather not apply, 3 = does rather apply, and 4 = does apply, with the 8th question designed as “yes” vs. “no” question and with the 10th question being an open one with the option to provide individual comments in a text box. The highest score on all scorable questions was 32, the lowest score was 4. The questionnaire was created in German and, for the purposes of presentation in this paper, translated to English (Additional file 1: Questionnaire 1). The second questionnaire, called *“Statements on experienced continuity of care”*, is a validated psychometric testing instrument, originally designed in the United Kingdom as an intervention tool, and has also been employed in other studies [18, 19]. The questionnaire is conceptualized in a way so as to pertain to all three components of perceived continuity of care. Patients rated all 17 statements on a five-point Likert scale with 1 = strongly disagree, 2 = disagree, 3 = neutral, 4 = agree and 5 = strongly agree. Hence 17 points is the minimum attainable score, 85 is the maximum attainable score [18, 19]. Like in other studies, high experienced continuity and coordination of care was said to exist when individual questionnaire scores exceeded or equaled 75 points [17]. For the purposes of this study, the authors of this questionnaire, Prof. King and Prof. Nazareth, gave their written permission to employ and to translate the questionnaire to German. The questionnaire was forward-translated by two independent radiation oncologists, and subsequently independently backward-translated to guarantee a high level of translation accuracy, as is recommended in guidelines [20]. The original version of Questionnaire 2 can be found in the Appendix as Additional file 1: Questionnaire 2.

Patients were either contacted via telephone to inquire about their willingness to participate in this study, then either invited for a brief clinical visit in our department, or the study documents incl. the questionnaires were sent via mail, or they were introduced to the study at the time of a routine clinical follow-up visit during the 3-month period March to May 2020. Upon having given written consent, patients completed the two questionnaires independently and at their own discretion. Study materials and filled-out questionnaires were subsequently returned via postal service or directly handed to the study office team. Patients not reached by phone were contacted via mail. No third attempt to approach was conducted to reach unresponsive patients. This project and its design were approved by the Swiss Cantonal Ethics Committee before study initiation (BASEC# 2021-00104).

### Data analysis

All clinical and treatment data were recorded in the spreadsheet program Microsoft® Excel® (Version 16.0). Descriptive statistics were calculated for all variables under study. Statistical differences between different groups of patients were assessed using the Fisher’s exact test due to the small sample size. Statistical significance was set at < 0.05. All statistical analyses were conducted using the Statistical Software Package STATA® (Version 16.1). Microsoft® Powerpoint® (Version 16.0) was used to compile graphics.

## Results

**Fig. 1:**
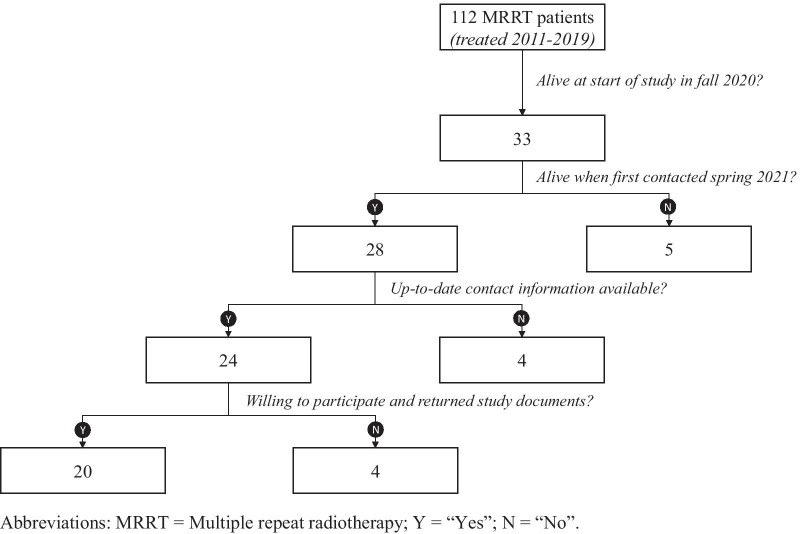
Consort diagram. Abbreviations: MRRT = Multiple repeat radiotherapy; Y = “Yes”; N = “No”

Between 2011 and 2019, 112 patients received at least five courses of RT at the Department of Radiation Oncology at USZ, totaling at 660 RT courses. At the time of initiation of this study in the fall 2020, 33 of these patients were still alive and were therefore included into this study at baseline. Five (15.2%) patients had died by the time they were contacted. Of the remaining 28 (84.8%) patients, all but one had an up-to-date address and contact details available and were approached; one patient had moved abroad without having provided a new postal address or telephone number and also did not respond to e-mail. Of all contacted patients, 24 (72.7%) initially indicated a willingness to participate in the study. During the second encounter, four (12.1%) patients refused to participate. Reasons for declining to participate were not wanting to be reminded of past therapies, wanting to live in the present, or having recently experienced health deterioration and therefore preferring to maximize time with family and friends rather than participating in a clinical research study. Hence, of the 24 (72.7%) patients who initially indicated a willingness to participate, 20 (60.6%) took part in this study (see Fig. 1 for a CONSORT diagram). As a result, objective metrics were calculated for all 33 (100%) patients, while PREMs were available for 20 (60.6%) patients.

### Patient and treatment characteristics

All 33 patients included in this study had at least one histologically verified cancer diagnosis. Twenty patients were male (60.6%), median age at diagnosis was 55 (range 32–75) years. The most common primary histology was lung cancer (n = 14, 42.4%). At time of data analysis, 28 (84.8%) patients were still alive. The median Eastern Cooperative Oncology Group Performance Status (ECOG-PS) before the last RT course was 1 (range 0–2). Patient characteristics are summarized in Table 1.

**Table 1:** Patient characteristics

Parameter	Data (n = 33 patients)
Age at diagnosis in years, median (range)	55 (32–75)
Female gender, n (%)	13 (39.4%)
*Primary tumor histology, n (%)*	
Lung cancer (SCLC and NSCLC)	14 (42.4%)
Breast cancer	3 (9.1%)
Colorectal cancer	3 (9.1%)
Sarcoma	3 (9.1%)
Urinary tract cancer	3 (9.1%)
Other^a^	7 (21.2%)
Alive at time of data analysis, n (%)^b^	28 (84.8%)
ECOG-PS before last RT, median (range)	1 (0–2)

ECOG-PS = Eastern Cooperative Oncology Group Performance Status; SCLC = Small cell lung cancer; NSCLC = None small cell lung cancer

^a^Includes malignant melanoma, head & neck, endocrine, primary brain entities as well as cancer of unknown origin

^b^Between time of official study initiation and time of data collection/analysis, 5 patients had died before being included in this study

The 33 patients received 210 RT courses, with a median of six (range 5–9) RT courses per patient. Forty-six (21.9%) prescribed RT courses had a curative, 164 (78.1%) a palliative intent. The most common irradiated tumor sites were brain metastases (n = 78, 37.1%) and bone metastases (n = 59, 28.1%). The median single dose was five (range 2–20) Gy, the median number of fractions was six (range 1–35), and the median total dose was 30 (range 6–70) Gy. A median of four (range 1–12) years elapsed between the first and last RT course. For an overview of compiled treatment parameters, see Table 2.

**Table 2:** Treatment characteristics

Parameter	Data (n = 210 courses)
Number of RT courses per patient, median (range)	6 (5–9)
*Treatment intent, n (%)*	
Curative	46 (21.9%)
Palliative	164 (78.1%)
*Treatment site, n (%)*	
Brain	78 (37.1%)
Bone	59 (28.1%)
Lung	37 (17.6%)
Primary	11 (5.2%)
Other^a^	26 (11.9%)
Single dose in Gy, median (range)	5 (2–20)
Fractions, median (range)	6 (1–35)
Total dose in Gy, median (range)	30 (6–70)
Time in years between first and last RT course, median (range)	4 (1–12)

RT = Radiation therapy

^a^Includes soft tissue and mediastinal metastasis

### Subjective measures for continuity and coordination of care

The median score for experienced, mostly relational, continuity of care obtained from the first questionnaire was 30 (range 25–32), which can be taken to be quite high given the highest score attainable when adding all scorable questions is 32. The question with the highest point score was asking about (1) the overall treatment experience at our radiation oncology department and the question with the lowest point score was question (7) on number of primary POs encountered during one’s treatment history. The proportion of patients having expressed agreement (3 or 4 points on the Likert scale) to questions one through seven and nine was n = 20 (100%). A large proportion of patients stated that they would opt for an additional course of MRRT if it was medically indicated (n = 18; 90%). A rather small proportion of patients (n = 8; 40%) reported to have made use of auxiliary supportive services available at CCCZ and only 4 (20%) patients made use of the comments box to provide further details on their care experience in the form of question (10). No significant differences were detected in subjective continuity of care when comparing the group of patients who had less or more than two POs over the years (*p* = 0.546). For a summary of PREMs from questionnaire 1, see Table 3a.

**Table 3:** (a) Summary of selected PREMs from questionnaire 1. (b) Summary of selected PREMs from questionnaire 2

Parameter	Total (n = 20)	< 2 POs (n = 8)	≥ 2 Pos (n = 12)	*p*-value *(Fisher)*
*(a)*				
Median score; points (range)	30 (26–33)	31 (28–33)	30 (26–32)	0.546
Pts with self-reported supportive service needs; n (%)	8 (40%)	3 (63%)	5 (37%)	N/A
Pts having made use of text box comments; n (%)	5 (20%)	2 (40%)	3 (60%)	N/A
*(b)*				
Median score; points (range)	75 (56–84)	74 (67–79)	75 (64–84)	0.456
Patients scoring ≥ 75; n (%)	11 (55)	3 (38)	8 (67)	0.362

Abbreviations: PREMs = Patient reported experience measures; Pts = Patients; PO = Primary oncologist

**Fig. 2:**
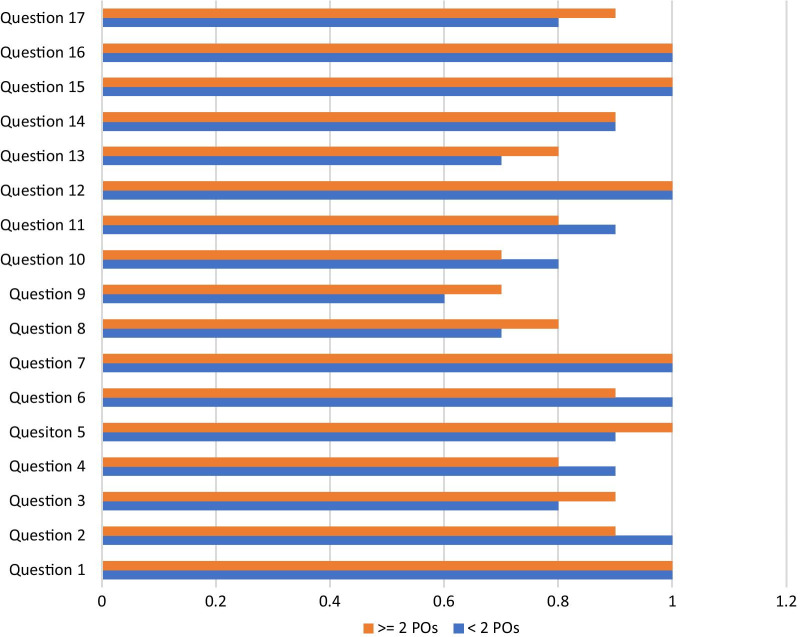
Proportion of patients with a positive response to Questionnaire 2. A positive score was taken to be a “4” or “5” on the Likert scale. Scoring for questions 4, 6, 9, 10, 11, 13 was reversed

The median score for experienced continuity of care obtained from the second questionnaire was 75 (range 56–84). There were eleven patients (55%) with a total score of ≥ 75 points. The question with the highest point score (95) was statement (7) on the support of close relatives, and the question with the lowest point score (63) was statement (9) regarding worries about relatives. There was only one patient (n = 1; 5%) having expressed agreement (4 or 5 points on the Likert scale) to all questions. No significant differences were detected in subjective continuity of care when comparing the group of patients who had less or more than two POs over the years (*p* = 0.456). There was also no statistically significant difference in achieving a ≥ 75 points score between the group of patients who had less or more than two POs over the years (*p* = 0.362). For a summary of PREMs from questionnaire 2, see Table 3b. Responses to the second questionnaire are also visually displayed in Fig. 2.

### Objective measures for continuity and coordination of care

A median of 6 years (range 3–13) elapsed between the first and the last follow-up visit at one of the clinical departments of the CCCZ. A total of 119 (56.7%) RT courses were preceded by an MDT discussion at the CCCZ. The median number of clinical departments involved in any given oncological patient care pathway was three (range 2–6). The oncology department had the follow-up lead for 13 (39.4%) patients and the radiation oncology department for twelve (36.4%) patients. For eight (24.2%) patients, other departments had the follow-up lead. The median number of involved POs at the radiation oncology department for the entire cohort of 33 patients was two (range 1–5). A summary of follow-up data is provided in Table 4.

**Table 4:** Follow-up characteristics

Parameter	Data (n = 33 patients)
Follow-up in years since first RT course, median (range)	6 (3–13)
No. of clinical departments involved, median (range)	3 (2–6)
*Department with follow-up lead, n (%)*	
Oncology	13 (39.4%)
Radiation Oncology	12 (36.4%)
Other^a^	8 (24.2%)
No. of POs in Radiation Oncology department, median (range)	2 (1–5)
No. of RT courses discussed in MDT, n (%)	119 (56.7%)

MDT = Multidisciplinary tumor board; RT = Radiation therapy; PO = Primary oncologist

^a^Includes dermatology, gynecology and nuclear medicine

When putting into perspective the examined objective measures for continuity of care with the subjective results from the validated questionnaire, there was no statistically significant difference between sub-groups. Of the patients scoring ≥ 75 points in the validated questionnaire, four (36%) were followed-up less than 6 years, while seven (64%) were followed-up for more than 6 years (*p* = 0.478). For six patients (55%) who scored more ≥ 75 points less than three clinical departments were involved in the process of care, while for five (45%) patients more than three played a role (*p* = 0.512). Of the patients scoring ≥ 75 points, the majority (n = 3, 73%) was followed-up by the radiation oncology department, while three (27%) were followed-up by other departments, yet also this difference was not statistically significant (*p* = 0.362). In four patients (36%) scoring ≥ 75 points, an MDT was never involved over the years, while in seven patients (64%) the MDT was involved at least once (*p* = 0.478). For a compilation of the different continuity of care measures, consult Table 5.

**Table 5:** Overview of different continuity of care measures

Parameter	Patients scoring ≥ 75 n (%)	*p*-value *(Fisher)*
*No. of follow-up years*		0.478
< 6	4 (36)	
≥ 6	7 (64)	
*No. of clinical depts involved*		0.512
< 3	6 (55)	
≥ 3	5 (45)	
*Dept with follow-up lead*		0.362
Radiation oncology	8 (73)	
Other	3 (27)	
*MDT involvement*		0.478
Never	4 (36)	
At least once	7 (64)	

Dept = Department; MDT = Multidisciplinary tumor board; PREMs = Patient reported experience measures; PO = Primary oncologist

## Discussion

Continuity and coordination of care at radiation oncology departments have not been studied systematically and in detail. Reasons may be that most patients receive RT only once in their life. While it always occurred that some patients received classical *re-irradiation*, i.e., RT to the same or an adjacent anatomical site and others *repeat irradiation*, i.e., RT to a different anatomical site, the population of MRRT patients only started growing recently. As MRRT patients are those characterized by having undergone the most complex therapy regimes, having been followed-up and having outlived their cancer diagnosis for several years, this fairly small, as highly selected, but growing group of cancer patients are those where continuity and coordination of care in radiation oncology are most relevant and therefore merit most attention.

Yet the importance of continuity and coordination of care has been acknowledged and studied in different oncological populations: Plate et al. [17] examined continuity of care in 231 breast cancer patients treated at two Swedish hospitals employing a validated questionnaire. The authors found that continuity of care was higher in the larger healthcare facility examined [17]. Husain et al. [9] assessed the continuity of care and supportive care needs in 116 advanced lung cancer patients in Canada with validated test instruments and concluded that patients with less unmet supportive care needs experienced higher continuity of care [9]. According to King et al. [18, 19] questionnaires and psychometric test interventions are the best way to measure and to improve continuity and coordination of care.

According to the subjective and objective measures employed in this study, the majority of patients in this cohort experienced high continuity and continuation of care along the informational, managerial and relational dimension. According to questionnaire 2, more than every second patient reported a high continuity of care according to the scoring scheme also employed in other studies (total score of ≥ 75 points in more than half of the patients). No significant difference was detected between the groups of patients who were seen by more or less than one PO over time. While it is widely accepted that PREMs are the most appropriate methodology to capture continuity of care, some authors have argued that they may also capture patient satisfaction, happiness or current state of health [21–23]. Yet also with respect to other objective measures such as length of follow-up, number of clinical departments involved, follow-up lead or MDT involvement, no significant difference in the number of patients with satisfactory subjective continuity and care scores could be detected. This finding may indeed be due to the small sample size of this study.

However, the good subjective scores are generally reinforced by the examined objective measures: Despite an academic university hospital setting, the median patient saw only two POs over a large number of RT courses over a long period of time (median of 6 RT courses and 2 POs per patient), indicating satisfactory relational and informational continuity of care. The fact that POs in our department have two organ subspecialties on average and that there are standard operating procedures placing great importance on recurring patients being managed by the same PO from initial consult to post-treatment follow-up may have contributed to this good result. This also seems to be reflected in the high scores of the questions regarding the relational aspect of continuity of care in questionnaire 1. The scores did not differ significantly between patients who had more or less than two POs. These results may be due to the fact that patients were followed-up for years at our department which is an integral part of CCCZ. In addition, in a radiation oncology department, besides the physicians, patients regularly and continuously engage with a large care provider team consisting of nursing staff and radiation therapy technicians who are present during all treatments, thus also contributing to the relational aspect of continuity of care.

Regular MDT meetings and case discussions are an integral part of cancer care in modern comprehensive cancer centers [24]. While there is a great variety in quality, format and frequency of MDT discussions, it is widely acknowledged that they improve quality and the managerial dimension of the continuity and coordination of care [25]. The evidence regarding the effect of MDT case discussions on outcomes is not yet conclusive, with some studies reporting improved clinical outcome and overall survival, while others merely found an association with higher recruitment to clinical trials [26, 27]. While the proportion of physicians regularly participating in MDTs as well as the number of patient cases regularly presented in MDTs is known to greatly vary, actual quantification of such figures is widely absent from the literature. In this cohort, only 57% of RT courses were discussed in MDTs, which falls short of the 80–95% sometimes required for comprehensive cancer center accreditation. This may present an indication of a lack of coordination among oncological disciplines, which again could have translated into a lower managerial and informational continuity of care. One reason for this rather low figure might be due to the fact almost 80% of RT courses administered in this patient cohort had a palliative intent with patients directly referred for symptom palliation. In these cases, an MDT discussion sometimes has to be traded off against a delayed treatment start. As a Cochrane review of fifty-one interventional studies on continuity of care concluded that it is difficult to assess the effectiveness of interventions and to actually pin down factors that may have contributed to a lower perceived continuity of care, one also needs to be cautious in this study to draw definitive conclusions when trying to correlated objective measures of coordination and subjective PREMs for the continuity of care [28].

Shortcomings of this study consist in its retrospective nature and the limited number of patients receiving MRRT. Given that treatment logs for the past decade were screened to identify patients having received MRRT, a larger assessment will only be possible when including multiple centers. Additional drawbacks of this study may originate from patients` chronic cancer disease and recall biases as well as from a long follow-up time with some patients having received their first RT treatment years ago. While the questionnaire response rate of effectively around 60% can be taken to be on the higher end of studies of this type, it also represents of risk of responses being upward-biased, as patients who have already died or declined to participate may have been those with unmet care needs and thus a lower perceived continuity of care. Though memory of treatment details may have been distant in some cases, the great majority of patients was in regular follow-up. Also, having completed at least five courses of RT and being able to extensively comment on this experience was a prerequisite of being able to participate in this study in the first place.

Yet, the strength of this study was the systematical assessment of the continuity and coordination of care in a large radiation oncology department of a tertiary center, using a validated questionnaire from the literature focusing on all three aspects of the continuity of care. Also, we placed particular importance on the relational aspect of the continuity of care, which is of central importance to cancer patients, by using an in-house developed questionnaire collecting patient-reported outcomes. Moreover, we hope to inspire other radiation oncology departments to conduct similar studies, so that pooled-data or multi-center analyses will be possible in the near future.

In conclusion, MRRT patients treated at our department and followed-up at the CCCZ over the past decade experienced high continuity of care by available measures. Further efforts should be undertaken to improve the informational, managerial and relational aspects of the continuity and coordination of care, for example, through increased PO continuity or the more regular use of MDTs in all patient cases, in order to provide even more patient-centered medical care and follow-up management.

## Supplementary Information

**Additional file 1.** Questionnaires 1 and 2 for assessing continuity of care. Questionnaire 1 is self-designed, questionnaire 2 is a validated tool from the literature.

## Data Availability

Data from electronic patient records cannot be disclosed.

## Abbreviations

BASEC: Business Administration System for Ethics Committees
CCCZ: Comprehensive Cancer Center Zurich
Gy: Gray
HIS: Hospital information system
MDT: Multidisciplinary tumor board
MRRT: Multiple repeat radiotherapy
PO: Primary oncologist
PREM: Patient reported experience measure
RT: Radiotherapy
USZ: University Hospital Zurich
