# Supplementary material for: Continuity and coordination of care in highly selected chronic cancer patients treated with multiple repeat radiation therapy
Source: Radiat Oncol. 2021 Nov 24;16:227. doi: 10.1186/s13014-021-01949-5 (PMC8611895; doi:10.1186/s13014-021-01949-5)
Supplement: Supplementary file 1 — Additional file 1. Questionnaires 1 and 2 for assessing continuity of care. Questionnaire 1 is self-designed, questionnaire 2 is a validated tool from the literature. [file 13014_2021_1949_MOESM1_ESM.docx]

**Appendix**

**Questionnaire 1**

| 1 | Since you were diagnosed with cancer, you have been treated numerous times in our department. Did you feel that you were being well looked after by the medical team? |
| --- | --- |
| 2 | Was it possible for you to build up a relationship of trust with your treating doctors over the months / years that you have been treated here? |
| 3 | In your opinion, did you have a permanent contact person in our radiation oncology department? |
| 4 | In your opinion, were you adequately informed about side effects in connection with frequent re-irradiation by the doctors in our department? |
| 5 | Were you satisfied with the medical follow-up and the discussion of treatment outcomes? |
| 6 | Overall, were you satisfied with the radiation treatment in our department? |
| 7 | All in all, did you find it a burden that the same medical contact person was not always responsible for you?^1^ |
| 8 | In addition to radiation therapy, have you made use of other services such as care by psycho-oncology, palliative care, social services, nutritional advice or other services?^1^ |
| 9 | Would you opt for another cycle of radiotherapy in our department if it was medically indicated? |
| 10 | Are there any other aspects / remarks relating to your treatment in the radiation oncology department that you would like to draw our attention to?^2^ |

Answer options on 4-point Likert scale: 1 = does not apply, 2 = does rather not apply, 3 = does rather apply, and 4 = does apply.

^1^ Scoring for this question is reversed.

^2^ “Yes” vs “No” answer required.

^3^ Text box for individual comments.

**Questionnaire 2 [18,19]**

| 1 | I have received enough time and attention from the cancer service. |
| --- | --- |
| 2 | I feel I am seeing the cancer service often enough. |
| 3 | I am getting consistent information about my illness from the health care staff. |
| 4 | I frequently have to chase up cancer service to get things done.^1^ |
| 5 | I have been well informed about what my treatment will involve over the next few months. |
| 6 | I feel out of touch with the cancer service.^1^ |
| 7 | I feel I am supported by the people closest to me. |
| 8 | I feel the people closest to me are able to cope with my illness. |
| 9 | I am worried about the emotional state of the people closest to me.^1^ |
| 10 | I feel I depend too much on the people closest to me.^1^ |
| 11 | I have received some misleading information from the cancer service.^1^ |
| 12 | I am satisfied that I have received a full medical examination with regard to cancer. |
| 13 | I am worried that some things have been overlooked.^1^ |
| 14 | I know I have a specific person at the hospital whom I can contact when I need to. |
| 15 | I know how to contact this person. |
| 16 | The last time I was in clinic, I think the medical staff had all my notes. |
| 17 | I feel I am able to manage between appointments. |

Answer options on 5-point Likert scale: 1 = strongly disagree, 2 = disagree, 3 = neutral, 4 = agree and 5 = strongly agree.

^1^ Scoring for these questions was reversed.
